# The R263K Dolutegravir Resistance-Associated Substitution Progressively Decreases HIV-1 Integration

**DOI:** 10.1128/mBio.00157-17

**Published:** 2017-04-04

**Authors:** Thibault Mesplède, Jing Leng, Hanh Thi Pham, Jiaming Liang, Yudong Quan, Yingshan Han, Mark A. Wainberg

**Affiliations:** a McGill University AIDS Centre, Lady Davis Institute for Medical Research, Jewish General Hospital, Montréal, Québec, Canada; b Division of Experimental Medicine, Faculty of Medicine, McGill University Montréal, Québec, Canada; c Department of Microbiology and Immunology, Faculty of Medicine, McGill University Montréal, Québec, Canada; University of KwaZulu-Natal

**Keywords:** R263K, human immunodeficiency virus, integration

## Abstract

Human immunodeficiency virus (HIV) infection persists despite decades of active antiretroviral therapy (ART), effectively preventing viral eradication. Treatment decreases plasma viral RNA, but viral DNA persists, mostly integrated within the genome of nucleated blood cells. Viral DNA blood levels correlate with comorbidities and the rapidity of viral rebound following treatment interruption. To date, no intervention aiming at decreasing HIV DNA levels below those attained through ART has been successful. This includes use of some integrase inhibitors either as part of ART or in treatment intensification studies. We have argued that using the integrase inhibitor dolutegravir (DTG) in similar studies may yield better results, but this remains to be studied. In treatment-experienced individuals, the most frequent substitution associated with failure with dolutegravir is R263K in integrase. R263K decreases integration both in cell-free and tissue culture assays. We investigated here how integrated DNA levels evolve over time during prolonged infections with R263K viruses. To investigate a potential defect in reverse transcription with R263K, the levels of reverse transcripts were measured by quantitative PCR. We measured HIV type 1 (HIV-1) integration in Jurkat cells over the course of 4-week infections using Alu-mediated quantitative PCR. The results show that R263K did not decrease reverse transcription. Prolonged infections with R263K mutant viruses led to less HIV-1 integrated DNA over time compared to wild-type viruses. These tissue culture results help to explain the absence of the R263K substitution in most individuals experiencing failure with DTG and support studies aiming at longitudinally measuring the levels of integrated DNA in individuals treated with this drug.

## IMPORTANCE

Antiretroviral treatment decreases plasma viral RNA, but HIV DNA persists for decades within infected cells. Studies of nonhuman primates have suggested that reducing retroviral DNA levels might represent a path to eradication. The integrase inhibitor dolutegravir is less susceptible than any other anti-HIV drug to the emergence of resistance in treatment-naive individuals. In treatment-experienced individuals, in contrast, rare cases of treatment failure were commonly associated with emergence of an R263K integrase substitution that confers low-level resistance to dolutegravir. It is unclear why this substitution is not more common in individuals experiencing failure with dolutegravir. We report here that R263K progressively diminishes the levels of integrated HIV-1 DNA in tissue culture over multiple cycles of infection. Our results help to explain aspects of the clinical efficacy of dolutegravir and suggest that this drug may be able to reduce HIV DNA levels within infected individuals compared to other drugs.

## INTRODUCTION

When antiretroviral therapy (ART) is available, prescribed, and used appropriately, it commonly suppresses plasma human immunodeficiency virus (HIV) RNA viral load below 50 copies per ml. In contrast, after an initial decrease in the first years of treatment, HIV DNA amount remains stable under ART (1). In some studies, the levels of viral DNA predict disease progression (2) and viral outgrowth (3) and correlate with residual viremia (4). To date, early treatment initiation is the only option to reduce these levels in humans (5). Adding the then newly discovered integrase inhibitor raltegravir (RAL) to successful ART did not longitudinally change the levels of total or integrated DNA in HIV-infected individuals, although it provided evidence for persistent replication (6). Indeed, in some treated individuals, an increase in two-long terminal repeat (2-LTR) circles—a by-product of integrase inhibition—was observed upon RAL intensification. Logically, in such individuals, HIV DNA levels should have reciprocally declined, but this was not reported (6). This apparent contradiction could be due to the fact that most of the integrated proviral DNA is replication incompetent (7), and thus, small changes in the levels of replication-competent DNA may not be easily measurable using current methods. In rhesus monkeys however, ART-treated simian immunodeficiency virus (SIV)-infected animals who additionally received a Toll-like receptor 7 (TLR-7) agonist termed GS-9620 benefited from a decrease in SIV DNA levels that translated into prolonged remission after treatment interruption (8). Clearly, reducing HIV DNA levels in individuals receiving ART may represent a path to viral eradication.

Currently, clinically approved integrase strand transfer inhibitors (INSTIs) are RAL, elvitegravir (EVG), and dolutegravir (DTG), while two other molecules, cabotegravir (CBG) and bictegravir (BTG), are currently under development (9). RAL, EVG, and DTG have demonstrated safety and efficacy and are now recommended for treatment initiation in newly diagnosed HIV-infected individuals in western countries in combination with reverse transcriptase inhibitors (RTIs). Virological failure in treatment-naive individuals who use RAL- or EVG-based therapy is associated with the emergence of mutations in the reverse transcriptase (RT) and/or integrase (IN) coding sequences (reviewed in reference 10). In contrast, no mutation in either RT or IN has been found in viruses isolated from treatment-naive individuals who experienced treatment failure under DTG-based treatment (10). In treatment-experienced INSTI-naive individuals, DTG failure can be associated with the emergence of the R263K integrase substitution, whereas INSTI-experienced individuals can experience failure with a variety of mutations commonly associated with RAL or EVG failure (11–13). Specifically, using DTG to treat INSTI-experienced individuals who had previously suffered from treatment failure with integrase substitutions associated with resistance against RAL or EVG (RAL/EVG) demonstrated that DTG efficacy was diminished in such individuals. For this reason, it was determined that such persons should receive 50 mg DTG twice a day (BID) rather than once a day (QD) (11–13). In addition, treatment failure in such individuals was associated with the presence of various RAL/EVG resistance substitutions that predated DTG use, most notably at positions Q148 and N155H (11–13). Intriguingly, previous exposure to RAL or EVG can also compromise the efficacy of DTG when the latter drug is used as a single antiretroviral agent, even when exposed individuals were previously free of treatment failure with either of the former drugs (14). As remarkable was the absence of R263K in viruses isolated from individuals who experienced failure with DTG monotherapy (14). Altogether, those results suggest that R263K preferentially emerges upon *ab initio* DTG pressure and also points toward the potential involvement of residual replication in the emergence of drug resistance (discussed in reference 10). We have extensively characterized the R263K substitution and showed that it decreases HIV integrase strand transfer activity at least in part by decreasing integrase DNA binding activity as measured in cell-free assays (15). In contrast to resistance pathways that develop with RAL or EVG, we found no compensatory mutation for R263K under DTG selective pressure (16–19). Molecular modeling further suggested that R263K indirectly alters the conformation of the integrase catalytic site through a cascade of amino acid interactions (15, 20). Short-term infections (24 to 72 h) have confirmed that R263K reduces the levels of integrated HIV type 1 (HIV-1) DNA compared to wild-type (WT) virus (15, 17).

Here we investigated how the defect in integration associated with R263K affects HIV-1 DNA levels during prolonged infections in tissue culture. Our results show that R263K does not affect reverse transcription and leads to a progressive decline in HIV-1 integrated DNA over time in prolonged infections. These findings may help to explain some of the clinical efficacy of DTG.

## RESULTS

### R263K does not decrease early and late reverse transcription.

**FIG 1:**
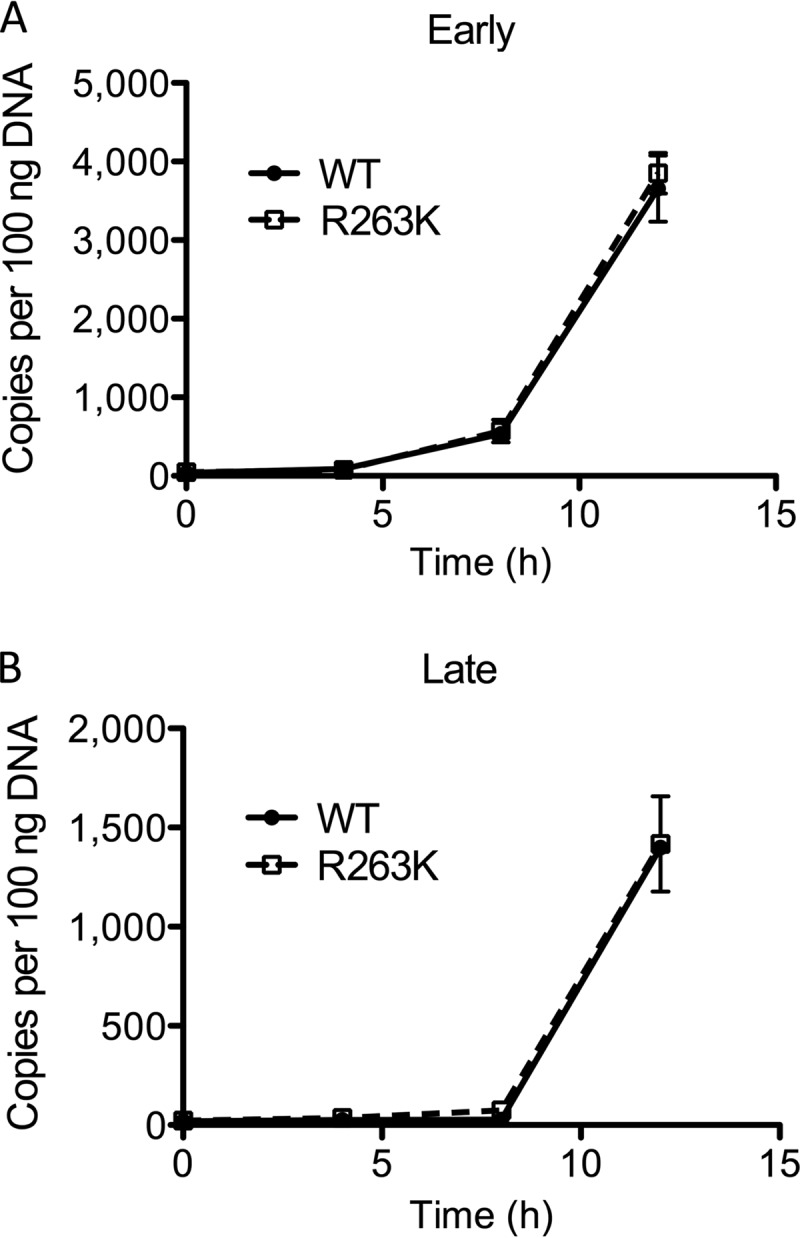
Kinetics of early (A) and late (B) reverse transcription during HIV-1 infections.

In order to exclude an effect of the R263K substitution on reverse transcription, we performed infections in Jurkat cells with the wild-type (WT) and R263K NL4-3 viruses and measured early and late reverse transcripts at various times after infection (Fig. 1). Primers and probes for early reverse transcripts were designed to quantify levels of double-stranded DNA (dsDNA) following minus-strand DNA transfer, whereas late reverse transcripts were produced concomitantly with the completion of reverse transcription (21, 22). The results show that WT and R263K viruses produced early and late reverse transcripts with comparable kinetics following infection.

### Prolonged infections with R263K-containing viruses result in gradual decrease in integrated DNA.

**FIG 2:**
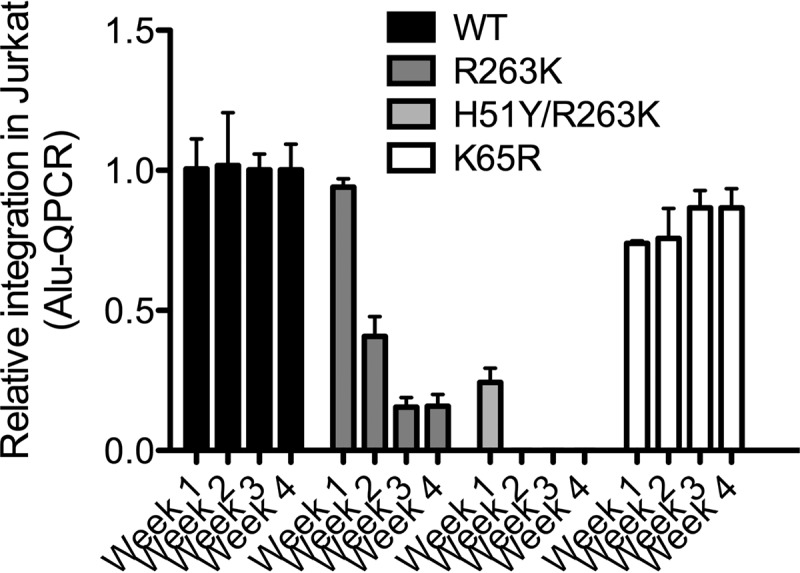
Weekly relative integration levels as measured by Alu-mediated quantitative PCR (QPCR).

**FIG 3:**
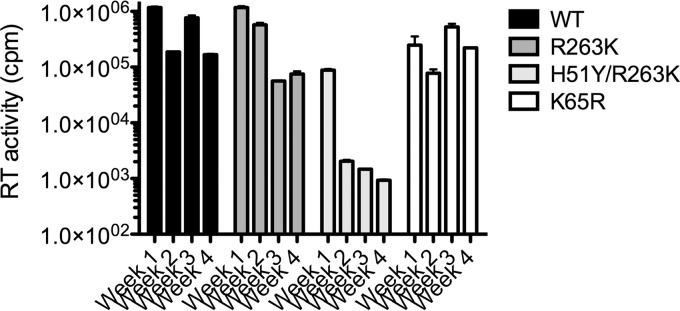
Weekly reverse transcriptase (RT) activity in culture fluids.

Given that integration is diminished by the R263K substitution in short-term infections (15, 17), we investigated the effect of prolonged infections on the levels of integrated DNA (Fig. 2). Prolonged infections were performed by transferring culture fluids from infected cells to uninfected cells at weekly intervals. H51Y is a substitution that can be selected under DTG pressure secondary to R263K and that further decreases integration compared to the latter mutation (17). We also used a virus bearing the K65R RT substitution as a control. After 1-week infections, the levels of integrated DNA were variously decreased by the presence of the various resistance mutations. More importantly, the R263K and H51Y/R263K substitutions resulted in a progressive decrease in integrated DNA between weeks 2 to 4 of infection. The levels of integrated DNA were decreased below the limit of detection after 1 week when the H51Y/R263K mutant was used. This progressive decrease was not observed with the K65R mutant. Measuring the RT activity in the cell-free culture fluids showed similar decreases in the production of infectious viral particles over time with the R263K and H51Y R263K mutants (Fig. 3).

## DISCUSSION

To date, DTG has been remarkably refractory to the emergence of drug resistance mutations when it has been used in first-line therapy. Whether this situation persists or whether a few individuals who are infected with viruses that can develop *de novo* resistance against this drug will eventually be identified is the subject of anxious scrutiny. An eventual *de novo* virological failure with resistance mutations may derive from preexisting rare polymorphisms, including E157Q and others (23, 24). In any case, to date, DTG has demonstrated an exceptional robustness against the emergence of drug resistance mutations in treatment-naive individuals. We have discussed the consequences of a potential impossibility of *de novo* resistance against DTG in treatment-naive individuals and suggested that this observation may be linked to the viral reservoirs (10, 25, 26). The development of HIV drug resistance follows a Darwinian process where drugs positively select resistant mutants. As such, the development of drug resistance mutations with DTG following preexposure to RAL or EVG suggests cryptic persistent replication under ART. Reciprocally, the absence of resistance mutations in RT and IN in treatment-naive individuals experiencing treatment failure with DTG suggests that residual viremia may be absent when this drug is used. Future studies are needed to investigate this.

In treatment-experienced individuals who experienced treatment failure while using DTG, the R263K substitution in integrase is the most common mutation reported thus far. We have extensively characterized this mutation in cell-free and tissue culture experiments and found that it diminishes integrase enzymatic strand transfer activity and HIV-1 DNA integration in cellular DNA in short-term infections (15). The present study further shows that prolonged infections with R263K-containing viruses result in a progressive decline in integrated HIV-1 DNA over time. This observation was even more pronounced with the H51Y/R263K combination of substitutions. This effect was specific, as infections with the K65R mutant did not result in diminished HIV-1 integration over time. Together with the fact that R263K did not decrease reverse transcription (Fig. 1), this suggests that the negative effect of R263K on HIV fitness is exclusively linked to the integration process. Importantly, our results show no differences in the amounts of both early (minus-strand DNA transfer) and late reverse transcripts that were produced during infections with WT or R263K viruses. In addition to decreasing integration levels, the R263K substitution may also alter integration sites. Changes in integration sites were observed when infections are performed in the presence of suboptimal concentrations of RAL or noncatalytic integrase inhibitors but also with the D116N catalytically inactive integrase mutant (27–29). In the case of the R263K substitution, however, these changes are unlikely to alter the establishment of or reactivation from latency, since we previously reported these processes to be unaffected by this substitution (30). Sequencing of R263K integration sites is under way.

The R263K substitution commonly results from a single nucleotide change, in most cases a single G→A transition. It is thus unclear why this substitution is not more commonly reported in individuals experiencing failure with DTG. One explanation is that R263K confers levels of resistance against DTG that are insufficient for treatment failure. Alternatively, R263K viruses may be so poorly fit that they may be outcompeted by WT strains upon virological failure. Results reported in the current study support the latter hypothesis. Indeed, progressive decreases in the levels of integrated DNA with the R263K mutant as shown in Fig. 2 may explain the disappearance of such virus *in vivo*. Further studies are needed to examine whether our results apply to cells that are known to contribute to the viral reservoir such as various subsets of CD4^+^ T cells (31). In the absence of longitudinal measures of cell-associated HIV DNA levels in individuals whose viruses have developed an R263K substitution under DTG-based therapy, the clinical relevance of our results is unknown. Whenever possible, we encourage physicians to closely assess and report clinical and laboratory parameters of such individuals.

Adding to the difficulty of monitoring such cases, we recently reported a transient R263K substitution in the proviral DNA of a chronically infected individual 4 weeks after initiation of DTG-based ART (32). This substitution was not detected in subsequent samples at either 24 or 48 weeks. Additional clinical studies are also needed to study the clinical impact of the R263K substitution on both viral load and viral reservoirs.

## MATERIALS AND METHODS

### Cells, viruses, and infections.

Jurkat human T-cell lymphoblasts and human embryonic kidney 293 (HEK-293) cells were obtained through the NIH AIDS Reagent Program, Division of AIDS, NIAID, NIH from Arthur Weiss and Andrew Rice, respectively (33, 34). Jurkat cells were propagated in Roswell Park Memorial Institute 1640 (RPMI 1640) medium supplemented with 10% fetal bovine serum (Gibco). Wild-type pNL4-3 was obtained through the NIH AIDS Reagent Program, Division of AIDS, NIAID, NIH from Malcolm Martin (35). The generation of the R263K and H51Y/R263K integrase and K65R reverse transcriptase mutants from pNL4.3 has been described previously (15, 17, 36). Viruses were produced by transfection of proviral plasmid DNA into HEK-293 cells, followed by isolation of viral particles as previously described (15). Jurkat cells were infected with 300 ng p24 cell-free viral particles per million cells for 1 h in a final volume of 1 ml after which cells were washed twice with phosphate-buffered saline (PBS) and cultured in 2 ml complete RPMI 1640 medium for 7 days. For prolonged infections, cells were pelleted each week by centrifugation, and 100-μl portions of cell-free culture fluids were used to infect one million uninfected Jurkat cells. These newly infected cells were then cultured for an additional week, and the same process was repeated after 7 days.

### Measuring early and late reverse transcripts.

Early and late reverse transcripts were measured as described previously (21, 22). Briefly, cellular total DNA was extracted using a DNeasy blood and tissue kit (Qiagen). DNA samples were quantified using a NanoDrop 1000 spectrophotometer (Thermo Scientific), and 65 ng DNA was amplified by PCR using the Platinum quantitative PCR (qPCR) SuperMix-UDG kit (Life Technologies, Inc.) and a Corbett Rotorgene 6000 thermocycler. The primers for early reverse transcripts were as follows: sense, 5′-GTGCCCGTCTGTTGTGTGAC-3′, and antisense, 5′-GGCGCCACTGCTAGAGATTT-3′. The probe for early reverse transcripts was 6-carboxyfluorescein (6FAM)-ACTAGAGATCCCTCAGACCCTTTT-MGBNFQ (minor groove binder nonfluorescent quencher) (Integrated DNA Technologies, Inc.). The primers for late reverse transcripts were as follows: sense, 5′-CCGTCTGTTGTGTGACTCTGG-3′, and antisense, 5′-GAGTCCTGCGTCGAGAGATCT-3′. The probe for late reverse transcripts was 6FAM-TCTAGCAGTGGCGCCCGAACAGG-MGBNFQ (Integrated DNA Technologies, Inc.). The primers and probes for early and late reverse transcripts were designed for the quantitative measurement of dsDNA produced immediately after transfer of minus-strand DNA and nearly complete reverse transcription, respectively. For both early and late reverse transcripts, the cycling conditions were as follows: (i) 50°C for 2 min; (ii) 95°C for 2 min; (iii) 50 cycles, with 1 cycle consisting of 95°C for 10 s, 60°C for 10 s, and 72°C for 30 s acquiring on the green channel; (iv) 25°C for 2 min.

### Measuring integrated DNA.

Integrated HIV-1 DNA was measured using a two-step Alu-mediated quantitative PCR as previously described (16). Briefly, 120 ng total cellular DNA purified as described above was amplified by PCR with the following primers: sense, 5′-GCCTCCCAAAGTGCTGGGATTACAG-3′, and antisense, 5′-GTTCCTGCTATGTCACTTCC-3′. A control reaction was performed in the absence of the sense primer. Both PCRs were subsequently used for quantitative PCR using the following primers: sense, 5′-TTAAGCCTCAATAAAGCTTGCC-3′, and antisense, 5′-GTTCGGGCGCCACTGCTAGA-3′. Cycling was performed on the same thermocycler as described above, as follows: (i) 50°C for 2 min; (ii) 95°C for 2 min; (iii) 50 cycles, with 1 cycle consisting of 95°C for 10 s, 60°C for 10 s, and 72°C for 45 s acquiring on the green channel; and (iv) 50°C for 2 min. The probe sequence for integrated DNA was FAM-CCAGAGTCACACAACAGAGGGGCACA-TAMRA (6-carboxytetramethylrhodamine).
