# Erratum for Mesplède et al., “The R263K Dolutegravir Resistance-Associated Substitution Progressively Decreases HIV-1 Integration”

**DOI:** 10.1128/mBio.01450-18

**Published:** 2018-09-25

**Authors:** Thibault Mesplède, Jing Leng, Hanh Thi Pham, Jiaming Liang, Yudong Quan, Yingshan Han, Mark A. Wainberg

**Affiliations:** a McGill University AIDS Centre, Lady Davis Institute for Medical Research, Jewish General Hospital, Montréal, Québec, Canada; b Division of Experimental Medicine, Faculty of Medicine, McGill University Montréal, Québec, Canada; c Department of Microbiology and Immunology, Faculty of Medicine, McGill University Montréal, Québec, Canada

## ERRATUM

Volume 8, no. 2, e00157-17, 2017, https://doi.org/10.1128/mBio.00157-17. One source of funding for our study was not properly acknowledged. The acknowledgment section of the manuscript should read:

We are grateful to Maureen Oliveira, Susan Colby-Germinario, and Estrella Moyal for technical assistance.
This project was supported by the Canadian Institutes for Health Research (CIHR) and Fonds de Recherche du Québec-Santé. Funding for aspects of this research was also provided by a grant from ViiV Healthcare (05983, project 106201).
The funders had no role in study design, data collection and interpretation, or the decision to submit the work for publication.

We regret to note that Mark A. Wainberg passed away in April 2017. Therefore, the corresponding author has been changed to Thibault Mesplède.

